# Outcomes of percutaneous coronary intervention in patients with coronary chronic total occlusions with versus without type 2 diabetes mellitus: A systematic review and meta-analysis

**DOI:** 10.1097/MD.0000000000008499

**Published:** 2017-11-10

**Authors:** Qiang Wang, Hao Liu, Jiawang Ding

**Affiliations:** a Institute of Cardiovascular Diseases, Yichang Central People's Hospital, Yichang, Hubei Province; b Institute of Cardiovascular Diseases, The Second Affiliated Hospital of Guangxi Medical University, Nanning, Guangxi, P. R. China.

**Keywords:** chronic total occlusion, major adverse cardiac events, mortality, percutaneous coronary intervention, type 2 diabetes mellitus

## Abstract

**Background:**

Nowadays, due to advanced techniques and well-trained interventionists in catheter labs, new scientific research has shown percutaneous coronary intervention (PCI) to be a safe treatment procedure in patients with chronic total occlusion (CTO). However, no study has systematically compared PCI outcomes in CTO patients with versus without type 2 diabetes mellitus (T2DM). Therefore, through this meta-analysis we aimed to systematically solve this issue.

**Methods:**

Between September 2016 and June 2017, the Cochrane Database of Randomized Trials, EMBASE, and MEDLINE databases were carefully searched for publications comparing PCI outcomes in CTO patients with versus without T2DM. Long-term (≥1 year) adverse clinical outcomes were considered the endpoints. Discontinuous data were analyzed by RevMan 5.3 whereby odds ratios (OR) and 95% confidence intervals (CIs) were the statistical parameters.

**Results:**

This analysis consisted of 1 randomized trial and 6 observational studies with a total number of 4571 patients with CTO (1915 patients with T2DM and 2656 patients without T2DM). Patients’ enrollment was between the years 1998 and 2015.

During this long-term follow-up (≥1 year), mortality was significantly higher in CTO patients with T2DM (OR: 1.56, 95% CI: 1.05–2.31; *P* = .03, *I*^2^ = 0%). Major adverse cardiac events (MACEs) and repeated revascularization were also significantly higher in patients with T2DM (OR: 1.30, 95% CI: 1.06–1.58; *P* = .01, *I*^2^ = 10%) and (OR: 1.30, 95% CI: 1.06–1.59; *P* = .01, *I*^2^ = 36%) respectively. However, myocardial infarction was not significantly different (OR: 1.01, 95% CI: 0.61–1.67; *P* = .96, *I*^2^ = 26%).

**Conclusion:**

During this longer follow-up period post-PCI, mortality, MACEs and repeated revascularization in CTO patients with T2DM were significantly higher compared with similar patients without T2DM. Nevertheless, whether this hypothesis is relevant or not should be confirmed in larger trials.

## 1 Introduction

To begin with, we should first know the definition of chronic total occlusion (CTO). CTO is the complete blockage of a coronary artery (normally ≥99% stenosis) for a duration of more than 3 months and it mainly affects patients with stable coronary artery disease (CAD). Even if this condition can easily be identified through coronary angiography, it is least preferred to be treated in interventional cardiology due to increased failure rates.^[1]^ In addition, treatment for CTO varied from 1 healthcare center to another and from region to region.^[2,3]^

Nowadays, due to advanced techniques and well-trained interventionists in catheter labs, new scientific research has shown percutaneous coronary intervention (PCI) to be a safe treatment strategy in patients with CTO. Safley et al^[4]^ further demonstrated PCI to be safe even in CTO patients with type 2 diabetes mellitus (T2DM). However, no study has systematically compared PCI outcomes in CTO patients with versus without T2DM. Therefore, through this meta-analysis we aimed to systematically solve this issue.

## 2 Materials and methods

### 2.1 Searched databases and searched strategies

The Cochrane Database of Randomized Trials, EMBASE, and MEDLINE databases were carefully searched for publications (English language) comparing long-term PCI outcomes in CTO patients with versus without T2DM by using the searched terms listed below:

1. chronic total occlusion, percutaneous coronary intervention, diabetes mellitus;
2. chronic total occlusion, coronary angioplasty, diabetes mellitus;
3. chronic total occlusion, PCI, diabetes mellitus;
4. CTO, percutaneous coronary intervention, diabetes mellitus;
5. CTO, PCI, and DM.

Reference lists of qualified articles were also checked for suitable publications.

This search was carried out by 2 independent reviewers (QW and HL) between September 2016 to June 2017 and included articles which were published from the year 2000 to 2016.

### 2.2 Inclusion and exclusion criteria

Inclusion criteria were:

1. randomized trials or observational studies comparing PCI outcomes in CTO patients with versus without T2DM;
2. studies reporting long-term (≥1 year) adverse outcomes as their clinical endpoints;
3. Exclusion criteria were:
4. any type of study except randomized trials or observational studies;
5. studies that did not include patients with CTO;
6. studies that did not compare adverse outcomes between T2DM and non-T2DM;
7. studies reporting short-term adverse outcomes (<1 year);
8. studies that were duplicated.

### 2.3 Types of participants

In this analysis, the participants were CTO patients with and without T2DM.

### 2.4 Endpoints and follow-ups

The endpoints were summarized in Table 1.

**Table 1:**
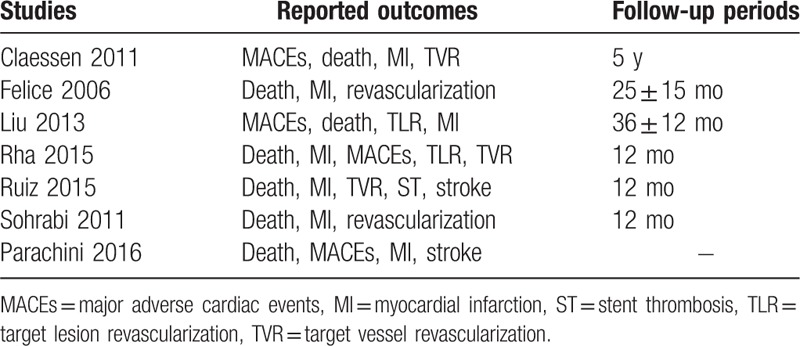
Reported outcomes and follow-up periods.

Endpoints included:

1. all-cause mortality;
2. myocardial infarction (MI);
3. major adverse cardiac events (MACEs) [which consisted of death, MI, and revascularization/stroke];
4. repeated revascularization (including target vessel revascularization and target lesion revascularization).

A longer follow-up period (≥1 year) was considered relevant in this analysis.

### 2.5 Data extraction

The same 2 reviewers who were mentioned above were involved in the data extraction process. Important information and data reporting the clinical outcomes, length of follow-up periods, type of study, periods of patients’ enrollment, total number of CTO patients with and without T2DM, the baseline features, and data reporting the total number of events that were observed in the experimental and control groups were carefully extracted and cross-checked. Any disagreement that occurred during this data extraction process was discussed and resolved by another reviewer (JD). The bias risk across trials (except observational studies) was assessed by the Cochrane Collaboration.^[5]^

In this analysis, PRISMA was used as the reporting guideline.^[6]^

### 2.6 Statistical analysis

Type of data to be analyzed: discontinuous.

Analytical software that was used: RevMan 5.3.

Analytical parameters: odds ratios (OR) with 95% confidence intervals (CIs).

Hypothesis testing: *P* value ≤.05.

Heterogeneity assessment:^[5]^ Cochrane Q statistic test and the *I*^2^ statistic test.

Significance of Cochrane Q test: *P* value of less or equal to .05 to be considered statistically significant. Any probability above .05 will not be significant statistically.

Significance of *I*^2^ statistic test: to measure inconsistency across the studies. An increasing *I*^2^ value signified an increased heterogeneity whereas a lower value indicated a low level of heterogeneity.

Sensitivity analysis: each study was excluded one by one and a new analysis was carried out each time and the main results that were obtained were compared for any significant difference.

Publication bias: visual assessment of funnel plot which was obtained.

Ethical approval: not applicable for meta-analysis.

Patients’ consents: not applicable for meta-analysis.

## 3 Results

### 3.1 Searched (databases) outcomes

One hundred twelve publications were obtained. After a careful assessment by the same 2 reviewers, 78 articles were eliminated. Thirty-four full-text articles were assessed for eligibility. Further eliminations were due to the following reasons:

1. case report (2)
2. studies not including patients without T2DM (control group) (8)
3. duplicates (17)

**Figure 1:**
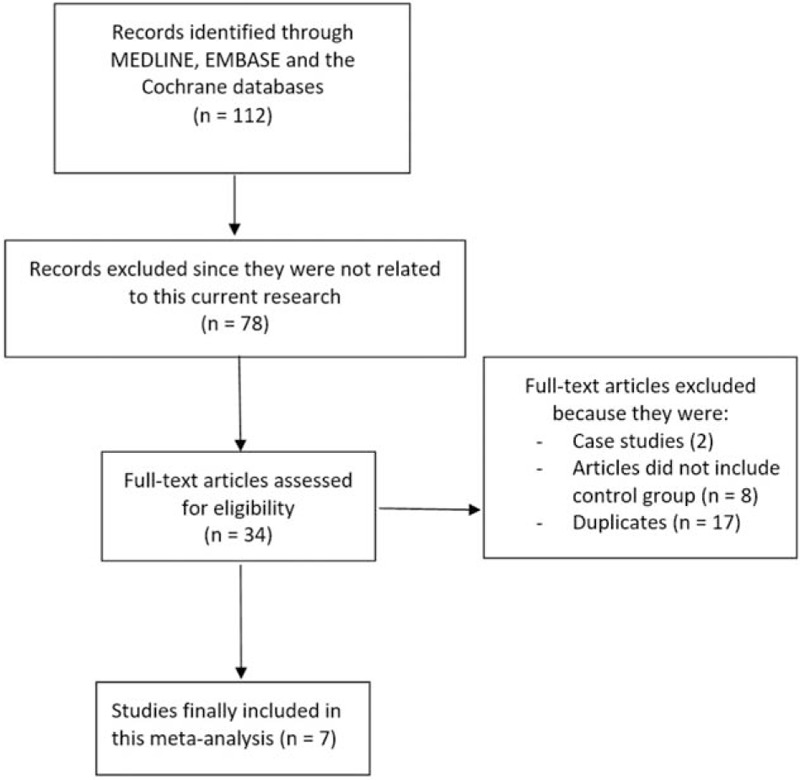
Flow diagram representing the study selection.

Finally, only 7 studies (1 randomized trial^[7]^ and 6 observational studies)^[8–13]^ were included in this analysis as shown in Fig. 1.

### 3.2 Main features of the studies which were included

The main features of the studies have been listed in Table 2. This analysis consisted of 1 randomized trial and 6 observational studies with a total number of 4571 patients with CTO (1915 patients with T2DM and 2656 patients without T2DM). Period of patients’ enrollment was between the years 1998 and 2015 as shown in Table 2. A bias risk grade B was allotted to the only trial available in this analysis.

**Table 2:**
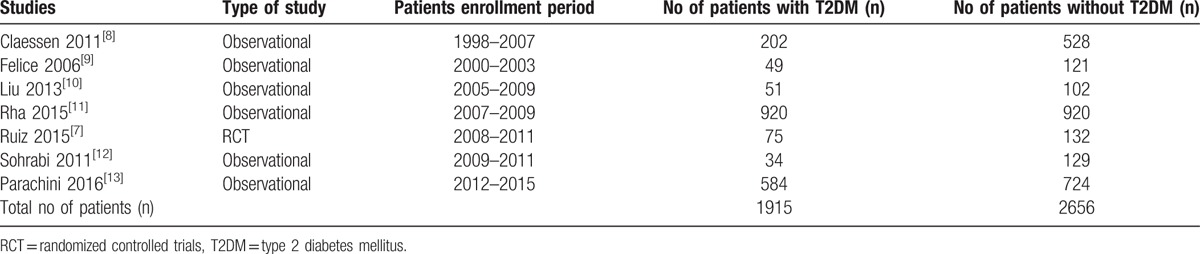
Main features of the studies which were included.

### 3.3 Baseline characteristics of the patients

As shown in Table 3, the mean age of the patients varied between 58.1 and 76.6 years. Majority of the patients were males compared with females in both the study and the control groups. Hypertension and dyslipidemia were more prominent among the patients with T2DM. Several studies reported a high number of smokers among the nondiabetic patients with a few exceptions as shown in Table 3. Overall, there was no significant difference in age between CTO patients with versus without T2DM; however, comorbidities were more prominent among patients with T2DM.

**Table 3:**
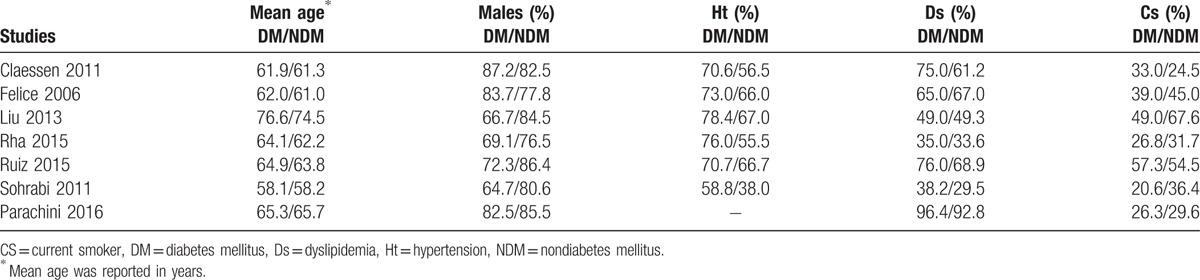
Baseline features of the studies which were included.

### 3.4 Long-term clinical outcomes

**Figure 2:**
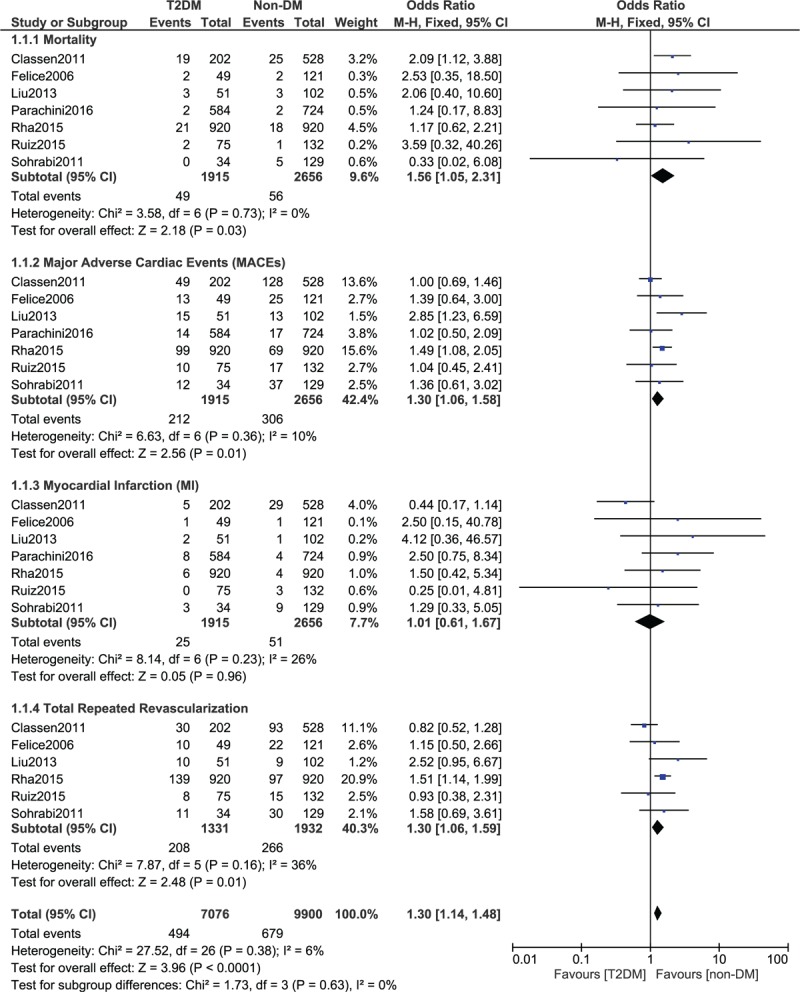
Long-term outcomes following PCI which were observed in CTO patients with versus without T2DM. CTO = chronic total occlusion, PCI = percutaneous coronary intervention, T2DM = type 2 diabetes mellitus.

This analysis showed that during a longer length of follow-up period (≥ 1 year), mortality was significantly higher in CTO patients with T2DM (OR: 1.67, 95% CI: 1.06–2.64; *P* = .03, *I*^2^ = 0%) as shown in Fig. 2. MACEs and repeated revascularization were also significantly higher in patients with T2DM (OR: 1.30, 95% CI: 1.06–1.58; *P* = .01, *I*^2^ = 10%) and (OR: 1.30, 95% CI: 1.06–1.59; *P* = .01, *I*^2^ = 36%) respectively as shown in Fig. 2. However, myocardial infarction was not significantly different (OR: 1.01, 95% CI: 0.61–1.67; *P* = .96, *I*^2^ = 26%).

The overall result has been listed in Table 4.

**Table 4:**
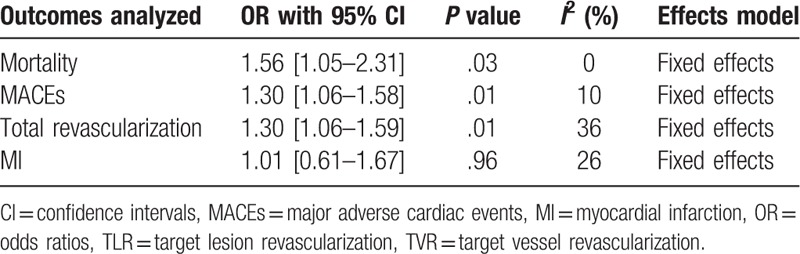
Results of this analysis.

### 3.5 Sensitivity analysis

Among the studies analyzing mortality, excluding each study one by one and carrying out a new analysis each time still showed mortality to significantly be higher in patients with T2DM except for study Classen2011 which when excluded, showed an un-significant result (OR: 1.29, 95% CI: 0.77–2.16; *P* = .33, *I*^2^ = 0%). Otherwise, consistent results were obtained when sensitivity analysis was carried out in all the other subgroups.

### 3.6 Publication bias

**Figure 3:**
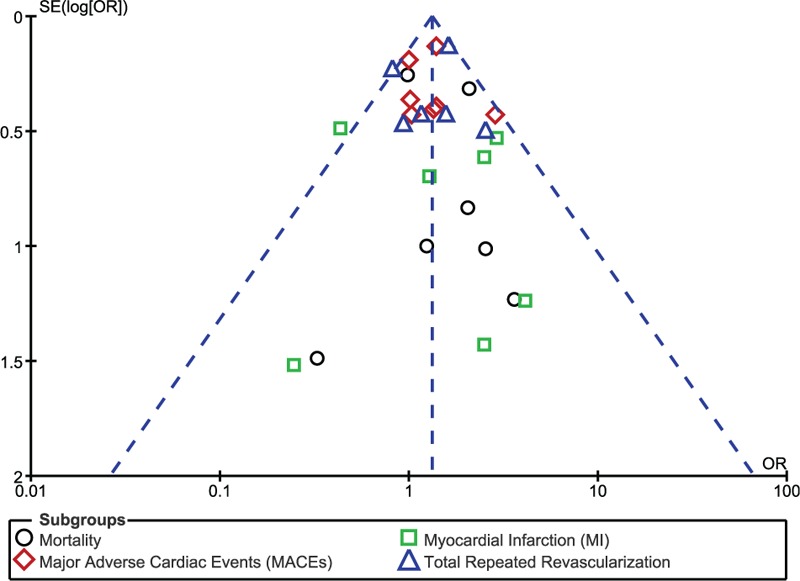
Funnel plot showing publication bias.

Publication bias across the studies was visually estimated by assessing the funnel plot which was obtained as shown in Fig. 3.

## 4 Discussion

In this analysis, we aimed to compare the long-term adverse clinical outcomes of PCI which were observed in CTO patients with versus without T2DM. Current results showed mortality, MACEs and repeated revascularization to be significantly higher among patients with diabetes mellitus.

Evolution in treatment of CTO due to a revolution in medical equipment in recent years has enabled high success rate among similar patients during PCI procedures.^[14]^ Even if randomized trials have rarely studied post PCI outcomes in diabetic patients with CTO, several observational studies have shown this invasive procedure to be safe in this particular subgroup of patients.

The Bypass Angioplasty Revascularization Investigation 2 Diabetes trial showed a higher mortality rate observed in CTO patients who were treated medically and the authors suggested that the presence of CTO might not always influence total death rate following revascularization in these patients.^[15]^

To further support this current analysis, Claessen et al^[16]^ demonstrated that the presence of CTO in a noninfarct related artery in patients with T2DM to be strongly associated with and could be considered an independent predictor of long-term mortality (5 years follow-up).

Moreover, Safley et al^[4]^ showed that PCI in T2DM patients with CTO was safe without causing any increase in MACEs or mortality when compared with matched patients without CTO. However, the authors clearly stated that there was no improvement in survival among these T2DM patients with CTO.

In this analysis, we have included mainly observational studies due to the lack of published trials. However, a recently published randomized trial, the CIBELES trial, showed different results compared with this analysis. CIBELES trial showed comparable outcomes in diabetic and nondiabetic patients with CTO following successful PCI.^[7]^ Mortality was also comparable between these 2 groups. However, the trial had a follow-up period of only 1 year, and involved only 75 patients with T2DM which was quite less to reach a conclusion.

Nevertheless, this analysis satisfied all the conditions to be qualified as a good meta-analysis in terms of robust results with low heterogeneity especially among the subgroup assessing mortality.

### 4.1 Novelty

This is the very first meta-analysis comparing the outcomes associated with PCI in CTO patients with and without T2DM. A low level of heterogeneity observed among the different subgroups could be another novelty of this analysis. In contrast to previous years, nowadays PCI is being considered safe in patients with CTO. Therefore, this analysis might provide new scientific knowledge and will help physicians predict prognosis in similar patients.

### 4.2 Limitations

Limitations in this analysis were the fact that a small sample size of patients were included. However, this was mainly dependent on the number of studies which were considered relevant in this analysis, as well as the total number of patients they included. Another limitation was the inclusion of observational data which might have been the source of heterogeneity during subgroup analysis. Moreover, different studies had different follow-up periods and this could be another possible limitation. In addition, the duration and type of antiplatelet drugs which were used could have had an effect on the results which were obtained. Not all the studies reported the duration of antiplatelet drugs.

## 5 Conclusions

During this longer follow-up period post PCI, mortality, MACEs and repeated revascularization in CTO patients with T2DM were significantly higher compared with similar patients without T2DM. Nevertheless, whether this hypothesis is relevant or not should be confirmed in larger trials.
